# Supplementary material for: The critical role of SENP1-mediated GATA2 deSUMOylation in promoting endothelial activation in graft arteriosclerosis
Source: Nat Commun. 2017 Jun 1;8:15426. doi: 10.1038/ncomms15426 (PMC5461500; doi:10.1038/ncomms15426)
Supplement: Supplementary Information — Supplementary Figures and Supplementary Table. [file ncomms15426-s1.pdf]

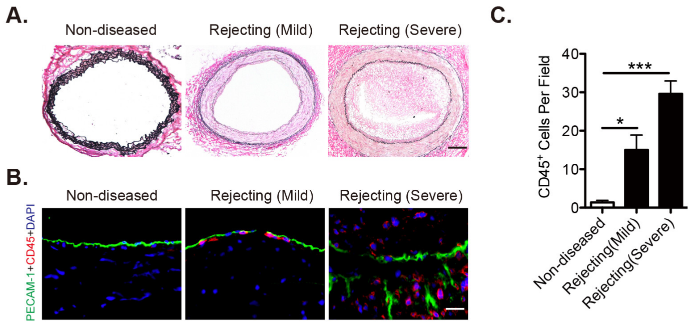

Supplementary Figure 1. Vascular inflammation in pathological remodeling in clinical GA. Human coronary arteries with GA or those without disease from non-transplanted hearts were collected. **(A)** Classical vascular remodeling in the graft vessel wall. Expansion of the neointima and media as well as pathological changes in the whole vessel wall is shown by Elastica–Van Gieson (EVG) staining. Bar represents 200 µm. **(B, C)** Inflammatory cell and vascular smooth muscle cell accumulation is demonstrated by immunofluorescence analysis of artery cross-sections that have been stained for the common leukocyte marker CD45 and the smooth muscle cell marker α-actin (SMA), including DAPI labeling of the nuclei. Representative images are shown in **B** with quantification data in **C**. Bar represents 50 µm. Data presented in **C** are the mean ± SEM from separate clinical specimens per group as indicated. *$P < 0.05$ and ***$P < 0.0001$; one-way ANOVA followed by Bonferroni test.

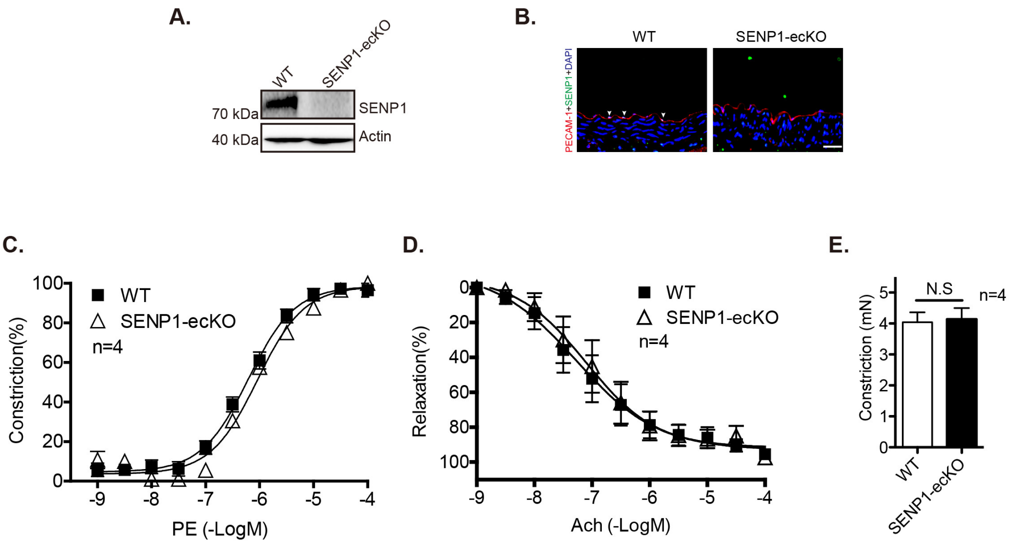

Supplementary Figure 2. Characterization of endothelial-specific SENP1 knockout mice. (A) Protein expression of SENP1 in MAECs from WT and SENP1-ecKO mice was determined by Western blotting. β-Actin was used as a loading control. **(B)** Endothelial SENP1 expression in the thoracic aorta of WT and SENP1-ecKO mice was determined by immunofluorescence analysis. PECAM-1 was used as a common marker for endothelium, and the nuclei were labeled with DAPI. Bar represents 50 μm. Data are pooled from 3 independent experiments. **(C)** WT and SENP1-ecKO aortas show a similar response to PE. Aortic rings from WT mice or SENP1-ecKO mice were contracted with PE at a full range of doses (10^{-9}-10^{-4} M). The percentage of constriction is shown. **(D)** WT and SENP1-ecKO aortas show a similar response to ACh. Aortic rings were precontracted with PE and then relaxed with ACh at a full range of doses (10^{-9} to 10^{-4} M). The percentage of relaxation is shown. **(E)** Endothelial SENP1 deficiency has no effects on vasoconstrictive response to KCl. Aortic rings were contracted with 50 mM KCl. Data are presented as the mean ± SEM from 4 animals per group that was averaged from 8 aortic rings per animal; **C** and **D**

comparisons were made by two-way ANOVA followed by Bonferroni post-test; **E** comparisons were made by unpaired *t* tests. N.S = not significant.

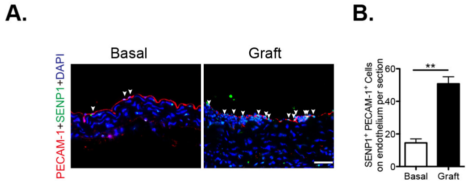

Supplementary Figure 3. Enhanced expression of endothelial SENP1 in mouse GA grafts. Endothelial SENP1 expression is examined by immunofluorescence analysis of coronary artery cross-sections that have been stained for SENP1 and the endothelial marker PECAM-1, including DAPI labeling of the nuclei. Representative images are shown in **A** with quantification data in **B**. Bar represents 50 μm.

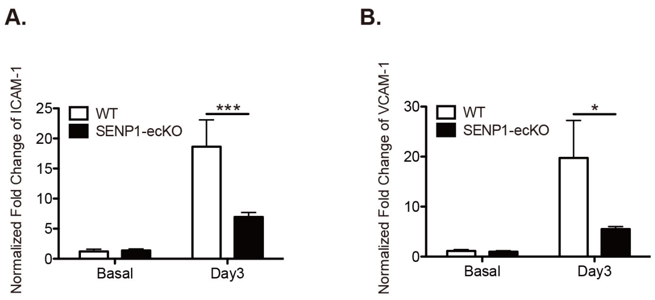

Supplementary Figure 4. Endothelial SENP1 deficiency results in reduced mRNA expression of endothelial adhesion molecules in the GA graft. (A, B) Quantitative fold change of ICAM-1 and VCAM-1 expression in grafts from WT and SENP1-ecKO mice before and 3 days after transplantation. Data were normalized to the WT graft before surgery and are shown as the mean ± SEM from at least 5 grafts. *$P < 0.05$ and ***$P < 0.0001$; two-way ANOVA followed by Bonferroni post-test.

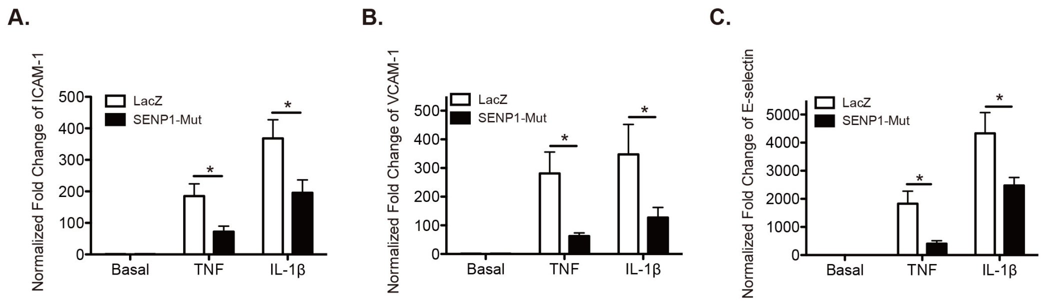

Supplementary Figure 5. SENP1 positively regulates mRNA expression of endothelial adhesion molecules in HUVECs. HUVECs were infected by Ad-SENP1-Mut or vector control (Ad-LacZ) viruses for 24 h and then stimulated by TNF or IL-1β for 4 h. The mRNA levels of *ICAM-1*, *VCAM-1*, and *E-selectin* before and after stimulation were detected by qPCR, and the normalized results are shown in **A**, **B**, and **C**, respectively. Data are reported as the mean ± SEM from at least 3 independent experiments. *$P < 0.05$; two-way ANOVA followed by Bonferroni post-test.

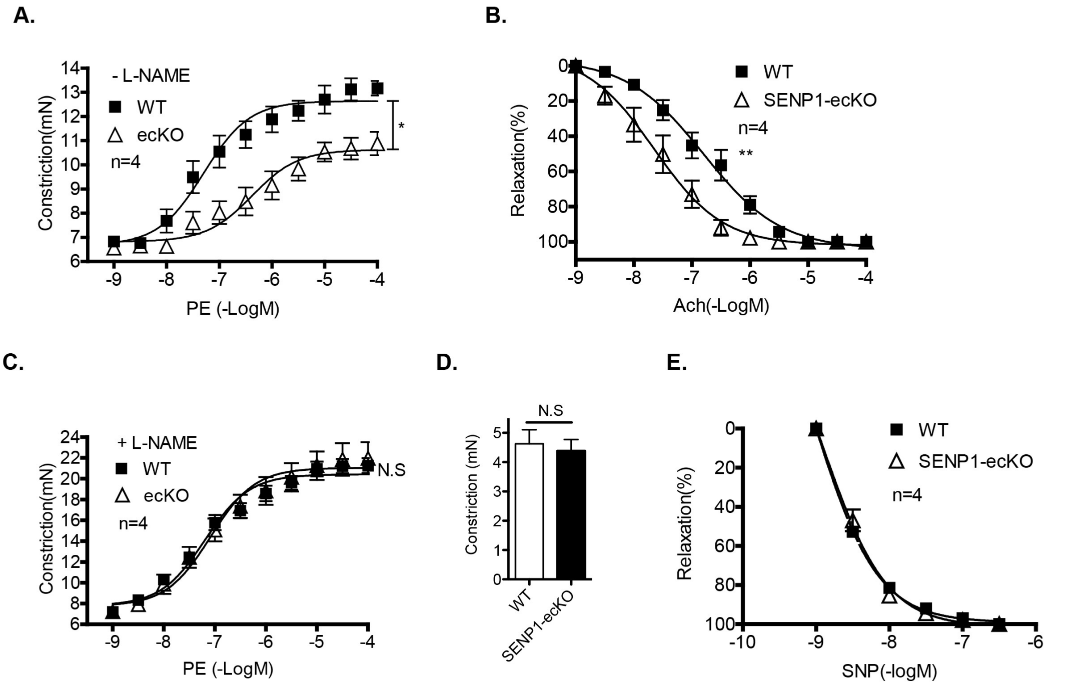

Supplementary Figure 6. Endothelial SENP1 deficiency prevents graft EC dysfunction. Aortas from WT or SENP1-ecKO male mice were transplanted to female B6 recipients, and the allografts were harvested at 1 week post-operatively. **(A)** SENP1-ecKO grafts show an attenuated response to PE. Graft aortic rings were contracted with PE at a full range of doses (10^{-9}-10^{-4} M). The constriction force (mN) is shown. **(B)** SENP1-ecKO grafts show an enhanced response to ACh. Graft aortic rings were precontracted with PE and then relaxed with ACh at a full range of doses (10^{-9} to 10^{-4} M). The relaxation force (mN) is shown. **(C)** WT and SENP1-ecKO grafts show a similar response to PE in the presence of L-NAME. Graft aortic rings were incubated with the NOS inhibitor L-NAME to inhibit the basal release of eNOS-derived NO prior to contraction with PE as in **A.** The constriction force (mN) is shown. **(D)** Endothelial SENP1 deficiency has no effects on graft vessel constriction in response to KCl. Graft aortic rings were contracted with 50 mM KCl. **(E)** Endothelial SENP1

deficiency has no effects on vessel relaxation to the NO donor drug SNP. Graft aortic rings were incubated with the NOS inhibitor L-NAME to remove basal NO synthesis prior to precontraction with PE as in **A** and subsequent relaxation with SNP at a full range of doses (10^{-9}-10^{-6} M). Data are presented as the mean ± SEM from 4 animals per group that was averaged from 8 aortic rings per animal; *$P < 0.05$ and **$P < 0.01$; **A**, **B**, & **D** comparisons were made by two-way ANOVA followed by Bonferroni post-test; asterisks indicate a significant contribution to the main effect of vasoreactivity by the mouse strain (WT or SENP1-ecKO); and **C** comparisons were made by unpaired *t* tests. L-NAME = N_{ω}-nitro-L-arginine methyl ester; and N.S = not significant.

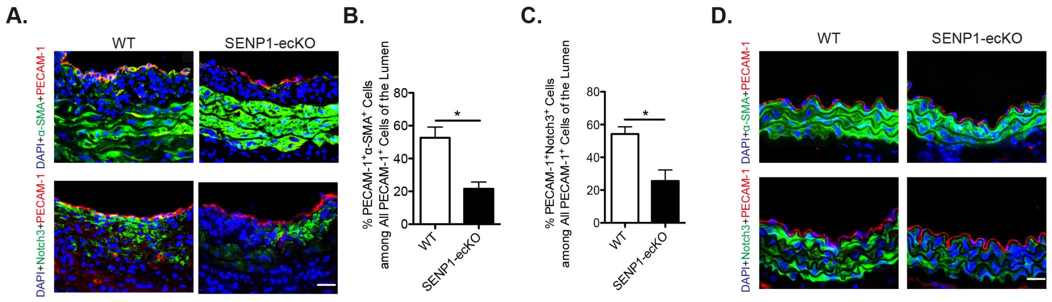

Supplementary Figure 7. Attenuated Endo-MT in SENP1-ecKO grafts at the middle stage. Grafts were analyzed by immunostaining with Endo-MT markers α-SMA and Notch3 with PECAM-1 to indicate the endothelium. The nuclei were stained with DAPI. Representative images of the middle stage are shown in **A,** with the percentage of lumen PECAM-1$^+$ cells that were α-SMA$^+$ or Notch3$^+$ in **B** and **C;** representative images of the early stage (3 days post-transplantation) are shown in **D**. Bar = 50 μm. Data in **B** and **C** are reported as the mean ± SEM from four independent grafts. *P < 0.05 compared with WT grafts; unpaired t test. Endo-MT = endothelial-to-mesenchymal transition.

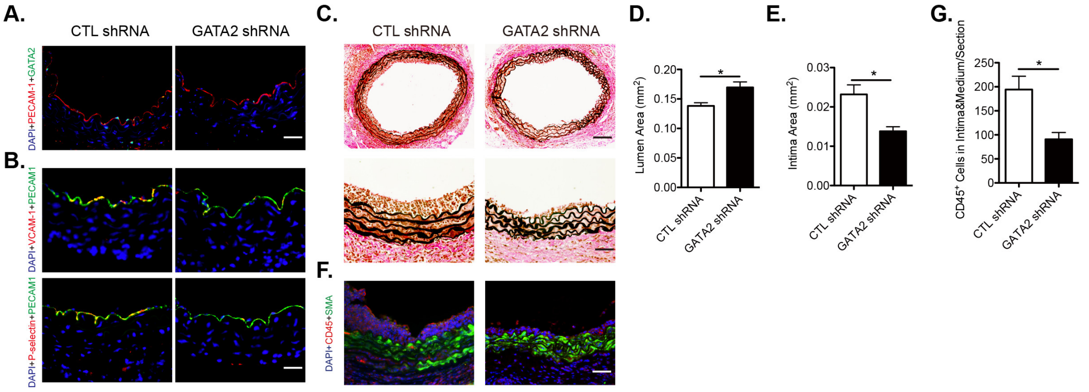

Supplementary Figure 8. Knockdown of endothelial GATA2 inhibits endothelial activation, inflammation, and GA. B6 male donor mice were injected with lentiviruses containing GATA2 shRNA or scrambled shRNA that was driven by the endothelial Tie2 promoter 1 week before transplantation into female B6 recipients. GATA2 shRNA significantly suppressed endothelial GATA2 expression in donor aortas **(A)**, which demonstrated reduced EC activation **(B)**, neointima formation **(C-E)** and inflammatory cell infiltration **(F-G)** after transplantation compared to the grafts treated with control shRNA. *$P < 0.05$; unpaired *t* test.

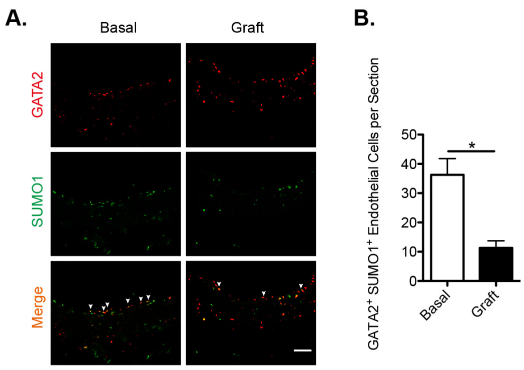

Supplementary Figure 9. Reduced endothelial GATA2 SUMOylation after transplantation in B6 grafts. GATA2 and SUMO1 were evaluated by immunofluorescence analysis in B6 non-transplanted aortas and grafts at three days after transplantation. Representative images are shown in A with quantification data in B. Bar represents 50 µm. Data are pooled from 3 independent experiments. $*P < 0.05$; unpaired t test.

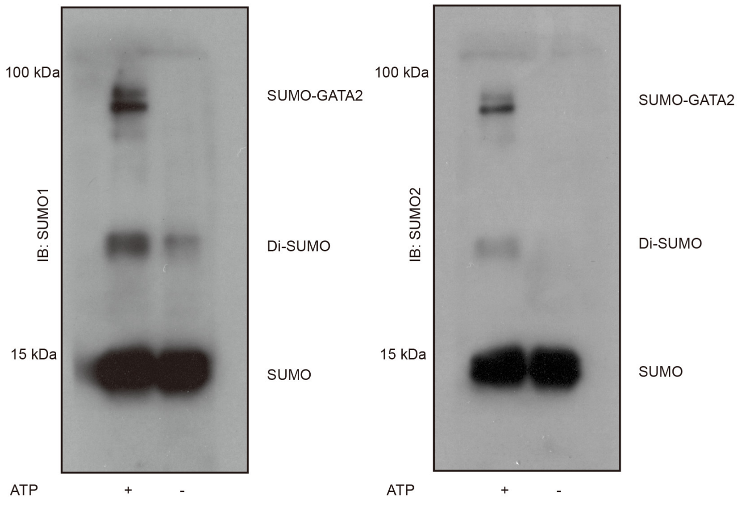

Supplementary Figure 10. GATA2 SUMOylation in an *in vitro* SUMOylation assay. The assay was performed using a SUMOylation assay kit as described in the Materials and Methods section. GATA2 SUMOylation was determined by incubating recombinant GATA2 with SUMO E1 and E2 in the presence or absence of ATP. The reaction mixture was subjected to Western blotting with anti-SUMO1 or anti-SUMO2 antibodies as indicated. Data are based on 2 independent experiments.

```
                                     *
Homo sapiens     SAARGEDKDGVKYQVSLTESMKMESGSPLRPGLATMGTQPATHHPIPTYP     250
Mus musculus     SAARGEDKDGVKYQVSLTESMKMESGSPLRPGLAAMGTQPATHHPIPTYP     250
Xenopus laevis   SVARGEDKDGVKYQVSLSESMKMEGGSPLRPGLATMGTQPATHHPIPTYP     250
Gallus gallus    ---RLEDKDSIKYQMSLSEGMKMEGGSPLRSSLAPMGTQCSTHHPIPTYP     224
Sus scrofa       --ARQEDKDSIKYQVSLSEGMKMESASPLRSSLTSMGAQPSTHHPIPTYP     237

                                                       *
Homo sapiens     NCQTTTTTLWRRNANGDPVCNACGLYYKLHNVNRPLTMKKEGIQTRNRKM     400
Mus musculus     NCQTTTTTLWRRNANGDPVCNACGLYYKLHNVNRPLTMKKEGIQTRNRKM     400
Xenopus laevis   NCQTTTTTLWRRNANGDPVCNACGLYYKLHNVNRPLTMKKEGIQTRNRKM     400
Gallus gallus    NCQTSTTTLWRRNANGDPVCNACGLYYKLHNVNRPLTMKKEGIQTRNRKM     373
Sus scrofa       NCQTTTTTLWRRNANGDPVCNACGLYYKLHNVNRPLTMKKEGIQTRNRKM     386
```

Supplementary Figure 11. Conservation of vertebrate GATA2 at the protein level. GATA2 protein sequence comparison among *Homo sapiens*, *Mus musculus*, *Xenopus laevis*, *Gallus gallus*, and *Sus scrofa*. Non-SUMOylated sites are black, and highly conserved SUMOylated sites (K222 and K389 marked by *) are in red.

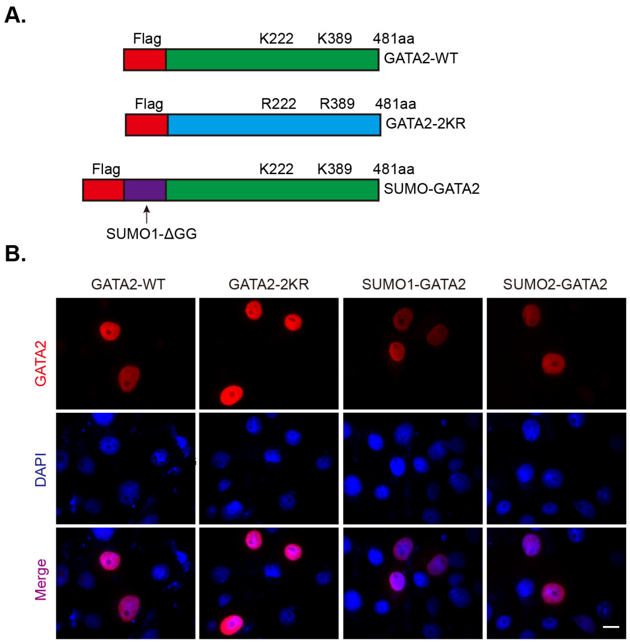

Supplementary Figure 12. SUMO modification had no effect on the nuclear localization of GATA2. (A) Schematic diagram for the expression constructs of GATA2-WT, GATA2-2KR, and SUMO-GATA2 fusion protein. The wild-type SUMOylation sites on GATA2 (K222 and K389) are indicated (top). aa, amino acid; and GG, Glycine-Glycine. **(B)** GATA2-WT, GATA2-2KR, SUMO1-GATA2, or SUMO2-GATA2 was transfected into COS-7 cells for 48 h. Localization of the various GATA2 proteins was determined by immunofluorescence staining using an anti-Flag antibody (GATA2). The nuclei were stained with DAPI. Bar represents 20 μm. Data are based on 3 independent experiments.

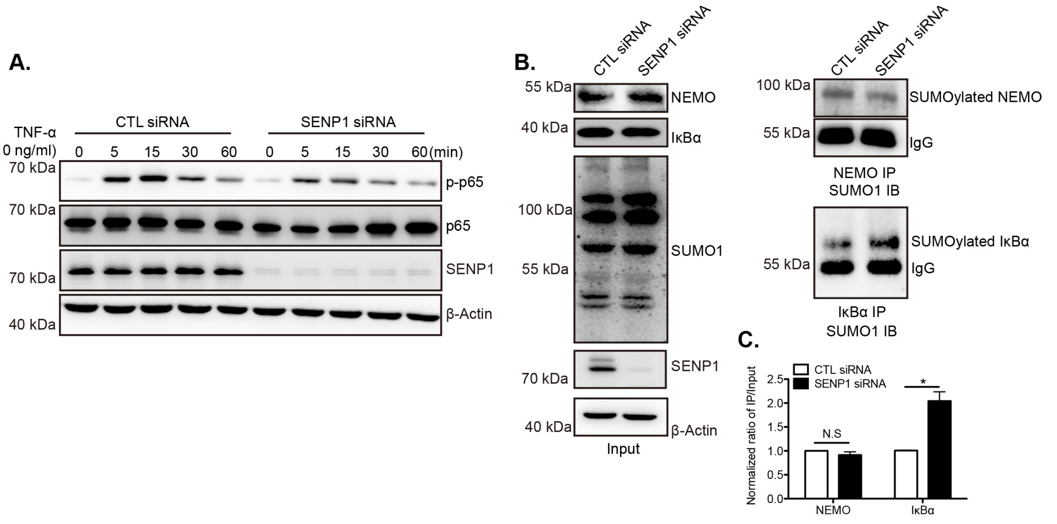

Supplementary Figure 13. SENP1 deficiency decreases NF-κB activity and enhances IκBα SUMOylation in endothelial cells. (A) Loss of SENP1 attenuates TNF-induced p65 phosphorylation. HUVECs were transfected by control or SENP1 siRNA for 72 h and then starved using serum-free medium for 4 h. After starvation, the cells were treated with TNF (10 ng ml^{-1}) for 0, 5, 15, 30, and 60 min in serum-free medium. p-p65, p65, SENP1, and β-actin were detected by Western blotting. **(B-C)** SENP1 deficiency increases IκBα SUMOylation. HUVECs were transfected by control or SENP1 siRNA for 48 h and subsequently treated with TNF for another 24 h. Protein lysates were harvested in the presence of 20 mM NEM. SUMOylated NEMO and IκBα were determined by IP with an anti-NEMO antibody or an anti- IκBα antibody followed by Western blotting with an anti-SUMO1 antibody. Input of NEMO, IκBα, SUMO1, SENP1, and β-actin was determined by anti-NEMO, anti-IκBα, anti-SUMO1, anti-SENP1, and anti-β-actin antibodies, respectively. Representative images are shown in **B** with quantification in **C**. NEM = N-ethylmaleimide. Data are pooled from

three independent experiments.

Fig. 5A

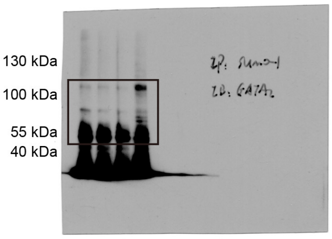

SUMOylated GATA2 and IgG

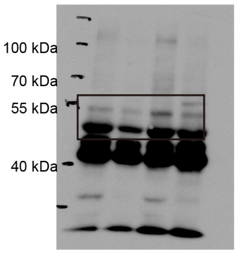

GATA2

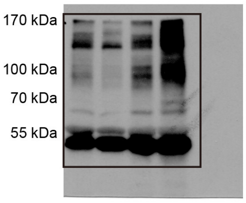

SUMO1

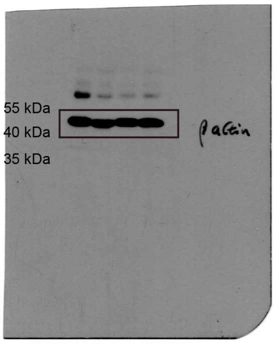

β-Actin

Supplementary Figure 14. Original blots shown in the main manuscript. Blots correspond to those shown in Figure 5A within the main manuscript.

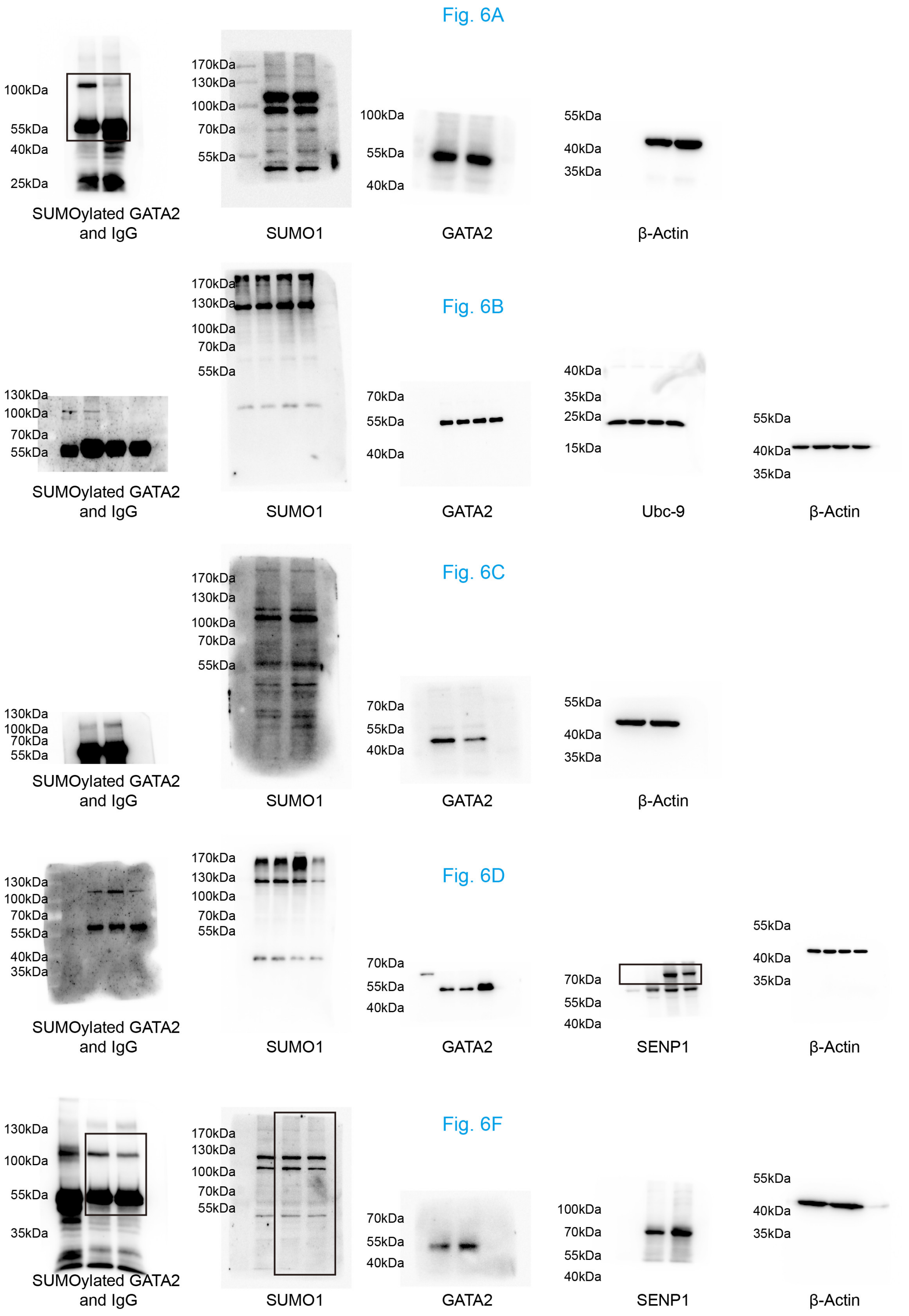

Supplementary Figure 15. Original blots shown in the main manuscript. Blots correspond to those shown in Figure 6A-D and Figure 6F within the main manuscript.

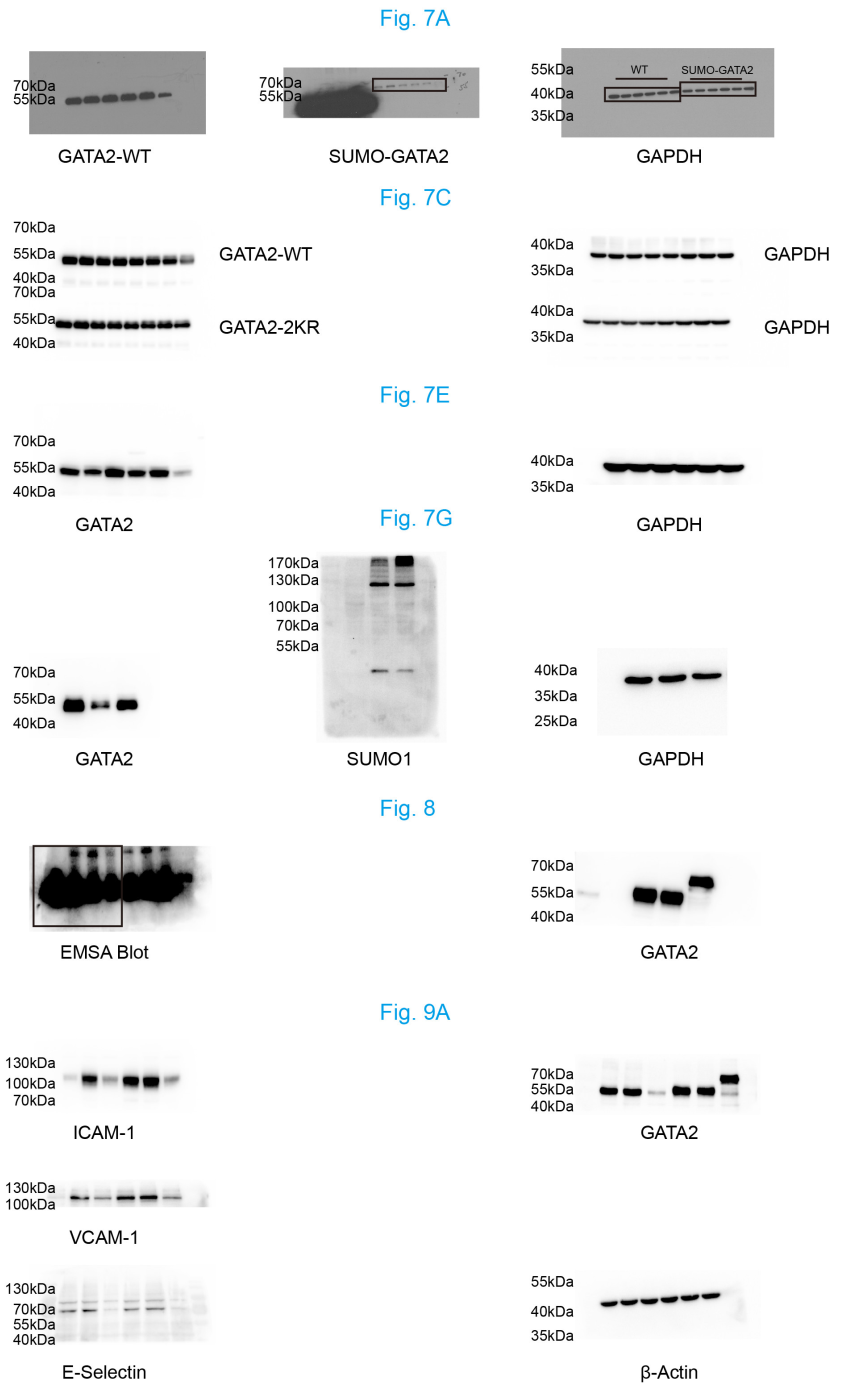

Supplementary Figure 16. Original blots shown in the main manuscript. Blots correspond to those shown in Figure 7A, Figure 7C, Figure 7E, Figure 7G, Figure 8, and Figure 9A within the main manuscript.

Supplementary Fig. 2

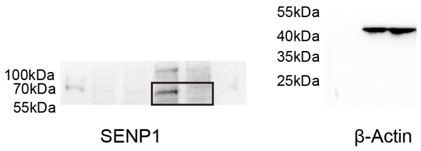

Supplementary Fig. 10

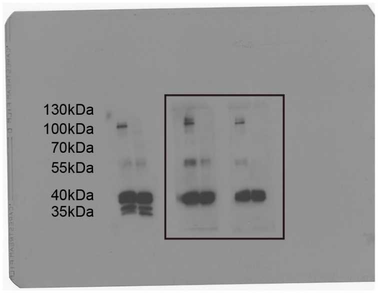

in vitro SUMOylation assay

Supplementary Fig. 13

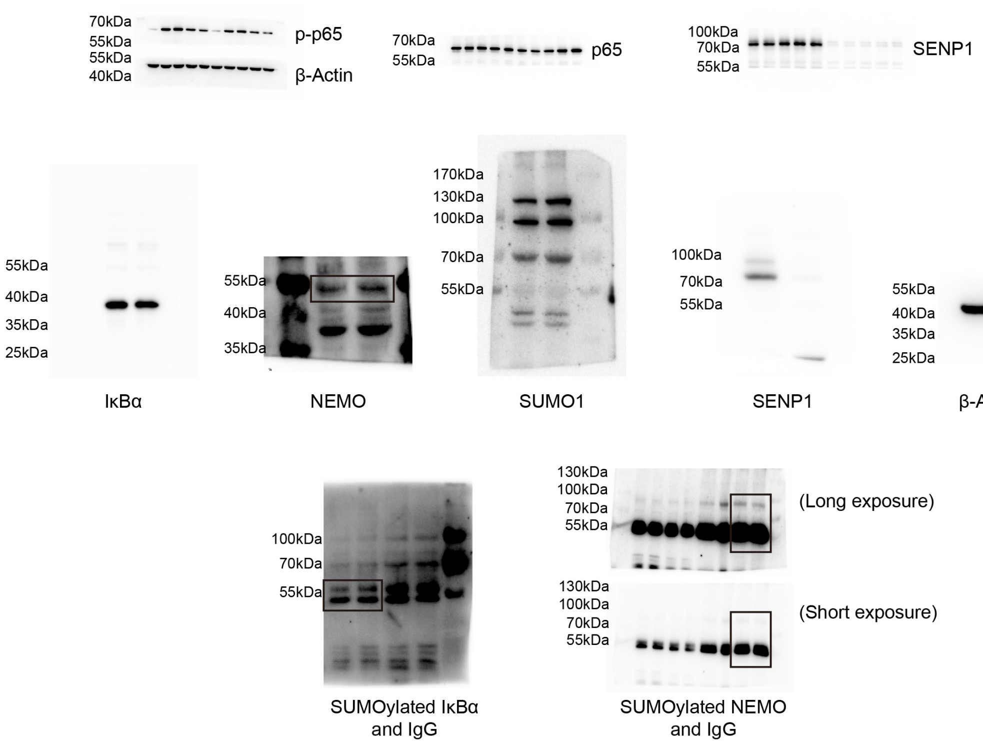

Supplementary Figure 17. Original blots shown in Supplementary Figure 2, Supplementary Figure 10, and Supplementary Figure 13.

Supplemental Table 1. Primer used in this study.

**Primer name**	**Primer sequence (5' - 3')**	
**Genotyping primers**	**Forward**	**Reverse**
Flox	AGGAAGGCATAGAAGTTACTCTA	GGTTGCTACTATAGTCAGACTG
Ve-Cadherin Cre	CCAGGCTGACCAAGCTGAG	CCTGGCGATCCCTGAACA
**Quantitative RT-PCR primers**		
Mouse ICAM-1	GTGATGCTCAGGTATCCATCCA	CACAGTTCTCAAAGCACAGCG
Mouse VCAM-1	GCCCACTAAACGCGAAGGT	ACTGGGTAAATGTCTGGAGCC
Mouse SENP1	CTGGGGAGGTGACCTTAGTGA	GTGATAATCTGGACGATAGGCTG
Mouse GAPDH	AGGTCGGTGTGAACGGATTTG	TGTAGACCATGTAGTTGAGGTCA
Human ICAM-1	GTATGAACTGAGCAATGTGCAAG	GTTCCACCCGTTCTGGAGTC
Human VCAM-1	TAAAATGCCTGGGAAGATGG	GGTGCTGCAAGTCAATGAGA
Human E-selectin	CAGCAAAGGTACACACACCTG	CAGACCCACACATTGTTGACTT
Human SENP1	CCCCATCATCACCACTCTGT	CTTTGATCCACAGCTCTGCC
Human GAPDH	CATTGCCCTCAACGACCACTTTGT	TCTCTCTCTTCCTCTTGTGCTCTTGC
**ChIP primers**		
Human ICAM-1 promoter	ATGAGCCTGGGTTCGAGTTG	AGGAAGGAAGCTGCGTGATC
Human VCAM-1 promoter	GGGAGATAGACCTGCACGTG	GGGCTGTGAGATTTAACCGC
Human E-selectin promoter	CCACTACACCCCGGCTAATT	TCTCTAGGCCAGGCATGATG
Human GAPDH promoter	TACTAGCGGTTTTACGGGCG	TCGAACAGGAGGAGCAGAGAGCGA
**GATA2 siRNA**	ACUACAGCAGCGGACUCUUTT	AAGAGUCCGCUGCUGUAGUTT
